# Opening the dialogue: Research networks between high‐ and low‐income countries further understanding of global agro‐climatic challenges

**DOI:** 10.1002/ppp3.17

**Published:** 2019-02-02

**Authors:** Stephanie Smith, Tirthankar Bandyopadhyay

**Affiliations:** ^1^ Sainsbury Laboratory Cambridge University Cambridge UK; ^2^ National Institute of Plant Genome Research (NIPGR) New Delhi India

**Keywords:** agronomy, fertilizer, India, international collaboration, nitrogen use efficiency, pollution, translational research, urea

## Abstract

Societal Impact Statement

In the modern world it has become increasingly urgent to balance human food security needs with environmental needs. These needs are not necessarily mutually exclusive, and can be synergistic. The Cambridge‐India Network for Translational Research in Nitrogen (CINTRIN) seeks to reduce nitrogen fertilizer overapplication (and the resulting environmental pollution) in Indian agriculture: a situation with various scientific and sociopolitical drivers, which equally have various sociopolitical and scientific solutions. By listening to the needs of local farmers and applying the knowledge and resources of global plant science research, achieving higher crop yields with less nitrogen is an achievable prospect for India.

## Graphical abstract

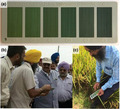

India is a country like no other. Widely tipped by politicians and economists to be a potential superpower by 2025, it boasts a space program and the second‐highest number of STEM graduates in the world (World Economic Forum, 2016). India has a youthful population which is highly technically literate: for example, 88% of households have a mobile phone (Registrar General of India, Census of India, 2011). However, rapid development here—as everywhere—comes at a cost.

As recently as the 1970s, India was consistently at risk of famine. Improvements in policy, government, transportation of food, overseas aid—and crucially, the Green Revolution—have meant that India is no longer routinely faced with wide‐scale famines of the sort which occurred during 1943 in the Bengal province, where millions succumbed to starvation. But the scepter of this dark past still looms large, and with a current population of 1.3 billion and growing, food security is still a high‐priority issue. This is apparent in the way that the Indian government subsidizes fertilizer: keen to ensure the highest yields possible, 1% of India's GDP is spent on fertilizer subsidies annually (Srivastava, 2017). This ensures farmers in India have plentiful nitrogenous fertilizer to use on their lands – in contrast to the situation in many low‐ and middle‐income regions such as sub‐Saharan Africa. (The World Bank currently classifies India as a `lower middle'‐income country and the United Kingdom as a `high'‐income country; World Bank, 2019)

But there is a catch. In most high–income countries, nitrogenous fertilizers represent a considerable proportion of an arable farmer's budget, and there is a strong financial incentive to adopt precision nutrition strategies. In these countries, agronomists, farmers and companies alike regularly perform analysis of optimum rates of nitrogen application, calculating the trade‐off point between the highest possible yields at the lowest possible rate of application. However, under the Indian policy, the financial incentive to limit nitrogenous fertilizer application is removed and over‐application is common: in some districts (particularly, in the Punjab region) rates of application may routinely exceed 200 kg per hectare (Tewatia & Chanda, 2017).

In India, nitrogenous fertilizer is mainly in the format of urea. The two main reasons for this are (a) ammonium nitrate‐based fertilizers, commonly used in Europe, are banned under anti‐terrorism laws in India to prevent their misuse in explosives; and (b) due to strong political and populist pressure, urea is exempt from the “Nutrient‐Based Subsidy” regulations introduced in 2010 to promote a more balanced use of fertilizer. This has biased fertilizer proportions strongly in favor of nitrogen (N) over other important (but less subsidized) nutrients such as phosphorous (P) and potassium (K), skewing ratios from the optimum NPK ratio of 4:2:1 to as high as 38:6:1 in the Punjab region (Jha, 2015).

Overapplication is further exacerbated by the policy of recommending “blanket application rates,” which do not take into account the existing nitrogen status of either the soil or plant. Culturally, there is also a commonly held belief among many Indian farmers that “greener is always better.” The origins of this belief are understandable: the correlation between N application and depth of leaf color is strong and perhaps more intuitively linear than the relationship between N application and grain yield, particularly at higher application rates.

This is a multifaceted problem for the environment, as overapplication of nitrogenous fertilizer (either organic or synthetic) can result in leaching into watercourses and eutrophication, impacting on aquatic life. Breakdown of nitrogenous fertilizer by soil microbes also releases several gases which can contribute to climate change and agricultural air pollution (methane, ammonia, and nitrous oxide). Agricultural air pollution is particularly insidious; ammonia combines with products from combustion to form fine particulate matter (also known as PM_2.5_) which can penetrate deep into lungs (Bauer, Tsigaridis, & Miller, 2016; Lelieveld, Evans, Fnais, Giannadaki, & Pozzer, 2015). It is this kind of agricultural air pollution which is the biggest cause of outdoor pollution‐related deaths, outstripping industrial air pollution. Furthermore, one cannot ignore the initial cost to the environment by production of fertilizer in the first place: more than 1% of the world's total energy use is consumed by the Haber–Bosch process used to fix atmospheric nitrogen into precursor.

To make matters worse, it has now emerged that overfertilization is defeating the very purpose it is meant to serve: productivity. Nitrogen only increases yield up to a point; after which adding more fertilizer becomes economically pointless or even detrimental through adverse effects on crop and soil health—for example, urea is toxic to crop roots at high concentrations (Cooke, 1962; Kelliher et al., 2005). In north Indian crop lands, farmers have been adding excessive fertilizer (sometimes more than four times the recommended level)—an alarming reality for India's food security (Anand, 2010).

On the face of it, the solution is simple: press Indian policymakers to reduce subsidies on urea, to a point where its use is considered more carefully. But (as is so often the case) the reality is more nuanced. With the pressures of food security so dominant in the government's priorities—and with many Indian farmers existing in a precarious financial state—India does not just need advice on how to reduce pollution, they need assurances that yields will be maintained and preferably even increase.

The Indian government is now increasingly looking at ways to achieve yields while reducing environmental cost. The Cambridge‐India Network for Translational Research in Nitrogen (CINTRIN), funded by the Newton–Bhabha Fund, aims to help with this. A collaborative project of several research institutions based in Cambridge (University of Cambridge, National Institute of Botany (NIAB), agriculture consultancy firm ADAS) and throughout India (National Institute of Plant Genome Research (NIPGR), New Delhi; International Crops Research Institute for the Semi‐Arid Tropics (ICRISAT), Hyderabad; Punjab Agricultural University (PAU), Ludhiana), the project aims to bring together the needs of local farmers and the resources of high‐level plant research. This is necessarily a two‐way dialogue: merely attempting to fit a “westernized model” of agronomy to the Indian agricultural system is an approach which is almost certainly bound to fail.

The strapline of CINTRIN is “A truly translational centre.” This is apt, as there is much to be translated: model species into crops, fundamental research into applied research, European‐style agronomic practices into Indian agronomic systems. But crucially, this is a bidirectional translation: the pipeline of knowledge flows both ways. The project is comprised of six work packages which encompass physiology, genetics, classical plant breeding and agronomy, bioinformatics, and expansion of the network, to include new partners who are tackling related issues.

A major part of increasing biological nitrogen use efficiency relies on identifying genetic targets to be translated into crop ideotypes to produce optimum yield on lower levels of nitrogen inputs. One factor which should be taken into consideration is that for at least the last 50 years in the west, the breeding of elite crop varieties has relied on lines selected from fields with high nitrogen inputs. This has led to impressive increases in yield, but this strategy runs the risk of selecting for lines which also have high N demands, and against genes which might confer higher yields at lower N levels. It is therefore possible that current elite varieties lack the genetic diversity to be a useful selection population for the identification of genes underlying optimum nitrogen use efficiency at low N.

One approach to circumvent this is to screen genetically diverse species. An example is the model grass *Brachypodium distachyon. Brachypodium *is related to many monocotyledonous cereal crops (in particular, rice) but as it has never been domesticated, it suffers no genetic bottleneck. Other benefits include a library of accessions available for screening, fast generation times, and a diploid genome with few repeating elements. Early screens in the United Kingdom show differing plasticity in developmental responses of *Brachypodium* accessions to different levels of nitrate (unpublished data); a RIL mapping screen is currently underway to identify the underlying genetic basis of this.

Meanwhile, CINTRIN researchers in India are screening the millet *Pennisetum glaucum* and the C4 millet *Setaria italica*. Millets are a group of C4 panicoids with significant genetic diversity, nutritional richness, natural stress tolerance, and other agronomically important characteristics. This makes them ideal candidates for regions and populations most vulnerable to food security and climate change such as south Asia and Africa (Bandyopadhyay Muthamilarasan & Prasad, 2017; Goron & Raizada, 2015). Importantly, millets are already part of the Indian staple diet; they are highly nutritious protein‐rich grains and an excellent source of nutritionally relevant minerals including iron, calcium, zinc, magnesium, phosphorous, and potassium (Hegde, Rajasekaran, & Chandra, 2005; Hulse, Laing, & Pearson, 1980; Muthamilarasan, Dhaka, Yadav, & Prasad, 2016). Millets promise affordable nutritional benefits—especially to those most vulnerable to malnutrition—while high temperature, drought, and salinity tolerance make them naturally suitable to many marginal lands (Calzadilla et al., 2013). Therefore millets can be regarded as climate change resilient crops with a potentially critical role in ensuring food security in south Asia and Africa. Investigations under the CINTRIN project are underway to observe and explain natural variation of biological nitrogen use in diverse foxtail accessions at the morphophysiological (nitrogen dose phenotyping, nitrogen partitioning studies) and genetic level (Genome Wide Association Studies/transcriptome analysis). This will potentially enable identification of elite millet germplasms and key physiological/genetic determinants with implications in crop improvement—a useful addition to the growing millet research community.

The relationship between carbon/nitrogen in plant development is also important; CINTRIN researchers are looking at photosynthesis, N uptake, and C/N allocation (primarily in wheat), in order to develop a better understanding of what constitutes optimal N use in key cereal germplasm. Genomics‐led pre‐breeding then incorporates any promising genetic targets into wheat, pearl millet, and/or foxtail millet. The pre‐breeding germplasm generated by this is tested for agronomic characteristics such as yield, tillering, and grain characteristics, and further evaluated for High Yield, Low Optimum N (HYLO) potential in field trials in both the UK and India.

CINTRIN also aims to provide resources for breeders and farmers in the field, and the first successes are starting to be delivered from this work ‐ with Indian scientists leading the charge. A team at the PAU (led by CINTRIN's Varinderpal Singh) has developed a version of a leaf color chart (PAU‐LCC) to help farmers in the Punjab region and beyond to better manage their N application strategy (Figure 1) (Ali, Thind, Sharma, & Singh, 2014; Singh et al., 2012). The LCC is intended to be a cheaper, more practical tool than the chlorophyll meters, such as Soil Plant Analysis Development (SPAD) meters, which are commonly used in high‐income countries to diagnose leaf N status; the PAU‐LCC retails for 100 Indian rupees (approximately $1.5 US dollars) and is beautifully low‐tech; all the farmer needs to do is align the chart with the leaves of their crop in full sunlight at specific growth stages, turn the chart over, and follow the tailored instructions on the back depending on which “green score” they recorded. CINTRIN has tested the PAU‐LCC with local smallholder farmers and early trials in the Punjab village of Bassian have been very promising. In 2017, the 60 rice farmers recruited for the trial reported that by using the LCC they achieved higher yields while saving on average 75 kg urea per acre; for some fields this represented a 50% saving in comparison to previous standard application rates. An additional welcome benefit was a reduction in insecticide use: overfertilization is known to increase the density of several pest species, including the local problematic rice pest, brown planthopper (Rashid et al., 2017).

**Figure 1:**
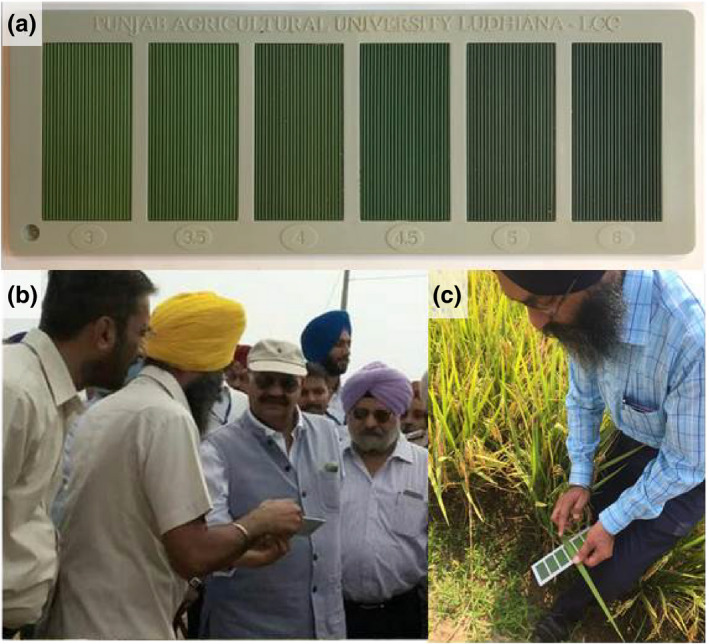
(a) The PAU‐LCC. Leaf spectral reflectance properties were used to produce a plastic strip with six different shades of green, these shades have been chosen to accurately correspond to the colors of actual leaves with empirically measured chlorophyll contents (within 5–10 SPAD units); (b and c) CINTRIN's Varinderpal Singh demonstrates the PAU‐LCC to farmers in the Punjab for use in trials

Finally—and potentially, most importantly—the project aims to foster collaboration between researchers in both countries, through training, exchanges of scientists between countries, outreach, and knowledge exchange between CINTRIN and other groups working on related issues, such as the Indo‐UK Centre for the improvement of Nitrogen use Efficiency in Wheat (INEW), Nitrogen Efficiency of Whole cropping System (NEWS), and the India–UK Nitrogen Fixation Centre (IUNFC). In particular, exchanges between scientists from low‐ and middle‐income countries and their counterparts have been praised by several of these centers as one of the most effective ways of building strong collaborations and a better understanding of the shared task (BBSRC Nitrogen Networking Meeting, group discussion).

Norman Borlaug was invited to India by geneticist Mankombu Sambasivan Swaminathan over 50 years ago; together they ushered in India's green revolution. Their approach is one we should seek to emulate: humble, collaborative, and willing to immerse oneself in the cultures and practices of different worlds. This renowned geniality was a factor in both Borlaug's scientific success and the affection of the Indian people for him; in 2006 he was awarded the Padma Vibhushan, the second‐highest civilian award India bestows.

In order to begin to build these relationships, individual researchers recognizing the value of local knowledge (and recognizing the limitations of their own knowledge) is the simplest first step. The academic network provides an excellent starting point for researchers from any discipline to make connections with institutions based in low‐ and middle‐income countries; furthermore, funding agencies are also increasingly promoting international collaboration in projects. But one also needs to go beyond academic connections to truly understand the needs of stakeholders; therefore potential stakeholders should be identified and consulted at earliest stages of a project. For CINTRIN, this involved Indian researchers such as Varinderpal Singh talking to hundreds of smallholder farmers to understand their concerns—engaging with stakeholders is both a useful way to improve the relevant outcomes of the project and, even more crucially, to build communication and trust.

Furthermore, interdisciplinary approaches are also being increasingly prioritized by funding bodies. For agriculturally focused projects such as CINTRIN, this is an excellent example where social science disciplines can make a key contribution. CINTRIN's sister project Transforming India's Green Revolution by Research and Empowerment for Sustainable food Supplies (TIGR2ESS) aims to define the requirements for a “second green revolution” in India by looking at policy agenda, academic and non‐academic stakeholders, NGOs, and industrial partnerships. It goes beyond the predominantly biological/agronomic focus of CINTRIN to attempt to understand and integrate broad social changes currently sweeping India, such as increasing urbanization and the way this has led to a greater proportion of female Indian smallholder farmers as men abandon farming for city‐based professions. TIGR2ESS aims to address multiple issues faced by Indian farming, in ways that are both technically and socially acceptable.

The question is often asked “what can high‐income nations do to help low‐ and middle‐income countries improve their food security?” To use a medical metaphor, we humbly suggest the answer is: listen to the patient and their unique symptoms before you prescribe the cure—and be prepared to learn something yourself!
